# Hemiarthroplasty in a patient with femoral neck fracture and pyoderma gangrenosum: a case report and review of the literature

**DOI:** 10.1186/s13256-019-2329-8

**Published:** 2020-01-14

**Authors:** Anna Antoni, Franz Trautinger, Thomas Heinz, Stefan Hajdu

**Affiliations:** 1 Department of Trauma Surgery, Medical University of Vienna, Währinger Gürtel 18-20, 1090 Vienna, Austria; 2 Karl Landsteiner Institute of Dermatological Research, Karl Landsteiner Society, Franziskanergasse 4a, 3100 St. Pölten, Austria

**Keywords:** Pyoderma gangrenosum, Hemiarthroplasty, Orthopedics, Trauma surgery, Pathergy

## Abstract

**Background:**

Pyoderma gangrenosum is a rare ulcerating skin disease of unknown etiology, making its coincidence with orthopedic trauma a rare challenge. Patients are at risk of progression of the existing lesions and development of new lesions upon skin injury when surgical procedures are performed. To our knowledge, this is the first report in the literature of disease unrelated surgery during active pyoderma gangrenosum.

**Case presentation:**

We present a case of femoral neck fracture in a Caucasian patient with concurrent pyoderma gangrenosum localized in the axilla. Hemiarthroplasty was safely performed after disease activity was reduced with systemic corticosteroids. Tissue-protective wound closure was used together with perioperative corticosteroids and antibiotics. No signs of pyoderma gangrenosum developed at the surgical wound site, and the axillary lesions showed constant improvement until healing with scar tissue.

**Conclusions:**

In our patient, the preoperative steroid treatment, perioperative antibiotics, and soft tissue protective surgical technique led to successful management of this rare coincidence.

## Background

The necessity to perform disease-unrelated surgery during active pyoderma gangrenosum (PG) is rare, putting surgeons in the difficult position of making decisions without underlying evidence. PG is a rare skin disease with unknown etiology. Brunsting *et al.* described the condition in 1930 and related it to staphylococci, but later research showed an autoimmune pathology to be more likely [1, 2]. In approximately 50% of cases, PG is related to trauma, surgery, malignancy, systemic inflammatory disease, and inflammatory bowel diseases such as ulcerative colitis and Crohn’s disease [3]. The pathergy phenomenon describes the development of additional lesions or progression of existing ones during active PG when skin integrity is interrupted [4]. It has been reported to occur at sites of intravenous lines, injections, and surgical wounds [5]. Pathergy puts the patient at risk for complications, resulting in recommendations not to perform any surgery on PG lesions; other authors recommend surgical management of PG ulcers in a less active period of the disease [6–8]. Systemic corticosteroids, especially prednisolone, and immunosuppressants such as cyclosporine A are the first-line medical therapies for PG. Other options include various local treatments, hyperbaric oxygen therapy, or debridement and skin grafting. Literature suggests that surgical treatment of PG ulcers can be successful after an initial phase of anti-inflammatory treatment [9, 10]. Based on the case of a patient with PG but no active lesions, general recommendations for their wound management, focusing mainly on reduced skin puncturing, have been published [11]. To our knowledge, there is no study or case report to guide a surgeon’s decision whether and how to operate on a PG-unrelated condition such as orthopedic trauma in patients with coincident active PG.

## Case presentation

**Fig. 1:**
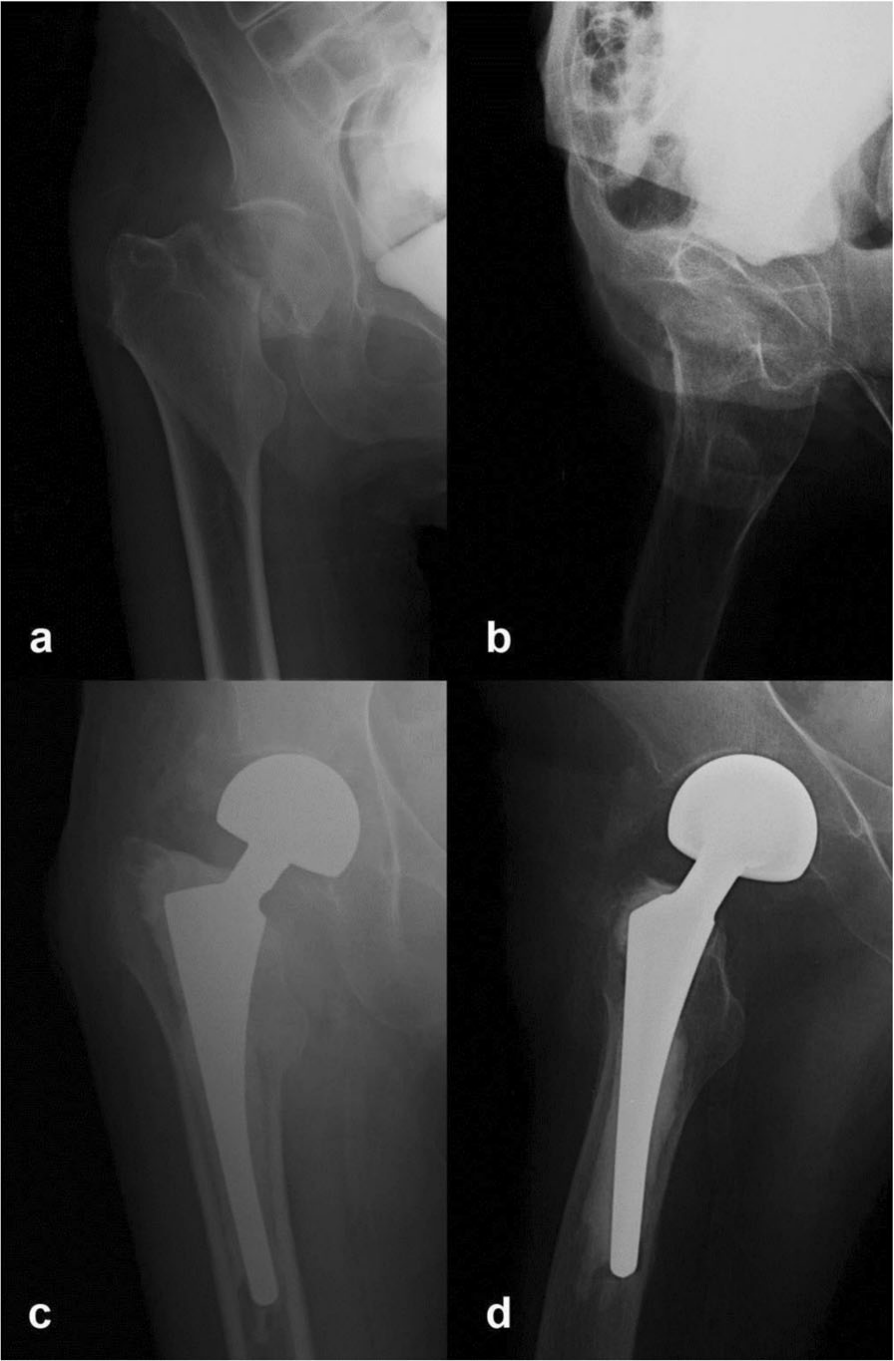
**a**–**d** Radiographs at time of diagnosis of femoral neck fracture, Garden IV, Pauwels III (**a**, **b**). Postoperative radiographs after cemented hemiarthroplasty (**c**, **d**)

**Fig. 2:**
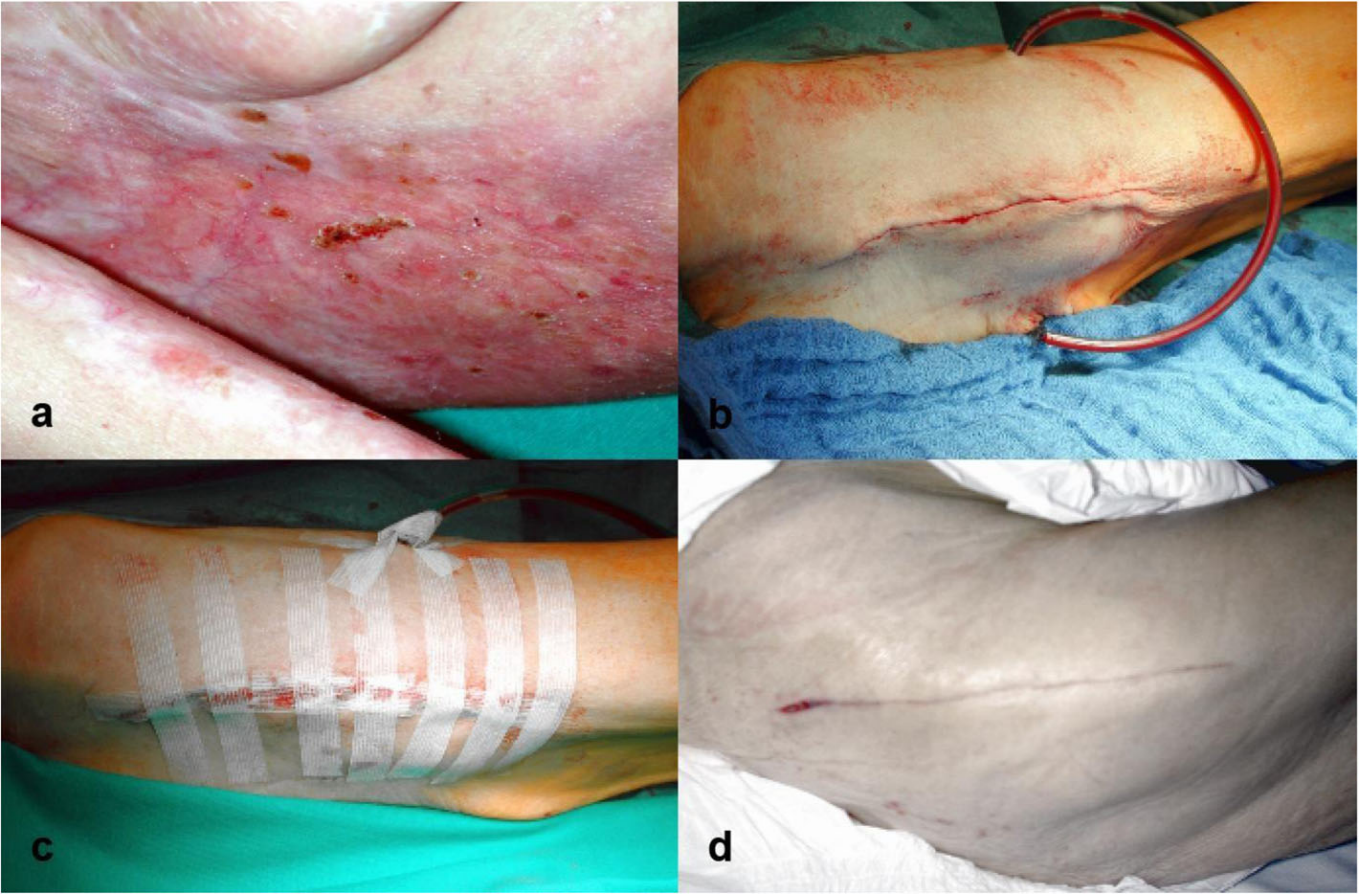
**a**–**d** Pyoderma gangrenosum lesion at time of surgery in the right axilla (**a**). Wound closure with subcutaneous absorbable polyglycolic acid (Dexon) sutures and Steri-Strips (**b**, **c**). Result 10 days postoperatively showing no pyoderma gangrenosum lesions, with only a minor wound dehiscence (**d**)

A 55-year-old Caucasian woman was referred to our level I trauma unit by the in-house dermatology department with a suspected right hip fracture after a fall at her home 3 days earlier. This previously healthy patient initially presented at the hospital and was admitted in a poor physical condition with painful ulcerating skin lesions, which covered the right axilla, lateral chest, and inner upper arm and discharged a purulent secretion. The patient described that the lesions had developed and worsened over the past few days, and she was initially referred to the dermatology department, where she was diagnosed with PG after clinical and histologic examination of the skin lesions. There were no systemic signs of infection at the time of presentation at the hospital. She was treated with local applications of octenidine dihydrochloride, paraffin gauze, and alginate dressings. Methylprednisolone treatment was initiated on the day of admission at a dose of 40 mg daily. Due to the reduced physical condition of the patient, who was in considerable pain at the site of the skin lesions, and preexisting general feebleness combined with underweight status, she was bedridden at the dermatology department. She did not report pain from her right hip until 3 days after admission to our hospital. The patient was then referred to our trauma department, where anamnesis, physical examination, and x-rays revealed a femoral neck fracture, Garden IV, Pauwels III (Fig. 1a, b). Due to the concurrent active PG lesions, the patient underwent temporary tibial traction. The patient’s lesions improved under care of the dermatology department (Fig. 2a), and there was no pathergy at the tibial traction site. Ten days after diagnosis of the femoral neck fracture and 13 days after the injury was sustained, surgery was performed with prophylactic moxifloxacin, which was continued at 400 mg daily. Hemiarthroplasty was performed according to the standard orthopedic technique with an anterolateral approach using bone cement (Fig. 1c, d). Only one subfascial wound drain was placed rather than the standard two or three drainages used at our department for femoral neck fracture arthroplasty. The wound was closed with subcutaneous absorbable polyglycolic acid sutures (Dexon®; Covidien, Minneapolis, MN, USA) and Steri-Strips® (3M, St. Paul, MN, USA) (Fig. 2b, c). Methylprednisolone was reduced to 20 mg daily after the tenth postoperative day.

Regular follow-up with clinical and x-ray examinations at our department showed no development of PG lesions at the surgical site (Fig. 2d). At final follow-up in our department 7 months after injury, the patient presented pain-free with unlimited range of motion of the right hip and full mobility. The PG lesions had healed with scar tissue, and she has had no further disease activity since her initial diagnosis of PG.

## Discussion

This unique case of orthopedic trauma during active PG suggests that disease-unrelated surgery using a soft tissue protective technique, preoperative immunosuppression, and perioperative antibiotics can be performed successfully. Due to the rarity of the disease, no larger studies on PG and either related or unrelated surgery are available, but the general rationale for operations during the course of PG is based on the principle of avoiding pathergy.

Case studies have shown that surgical management of PG lesions without prior medical treatment led to worsening of the ulcers. In most of these reported cases, the PG was misdiagnosed as wound infection and treated with antibiotics and surgery alone. Antibiotics consistently showed no effect, and surgery resulted in worsening of the lesions when no appropriate PG treatment was initiated [6, 12]. Some authors even reported cases of lethal complications due to pathergy after surgical treatment of ulcers [9]. Evidence for the effectiveness of soft tissue protective surgery in PG is described in the case report by Long *et al.*, who compared two surgical wound closure techniques in a patient with a medical history of PG. The 61-year-old woman underwent bilateral hip arthroplasty, but unlike our patient, she had no PG lesions at the time of surgery and received low-dose prednisolone as a maintenance treatment. The first arthroplasty was performed using interrupted silk sutures to close the skin and led to multiple ulcers at the suture entry/exit sites. Therefore, the authors used continuous subcutaneous Dexon® sutures and Steri-Strips® for skin closure of the second arthroplasty. After this surgery, only two smaller and less florid ulcers occurred. On the basis of this experience, the authors concluded that soft tissue protective surgery reduces the risk for pathergy in patients with PG [11]. She *et al.* described a case of avascular necrosis of the femoral head and a femoral neck fracture in a patient with a history of PG. They successfully performed total hip arthroplasty with perioperative corticosteroid therapy and did not describe any specific soft tissue protective management [13].

Several reports have shown surgical management of PG lesions such as debridement and skin grafting to be successful if performed in a less active phase of the disease after initial treatment with immunosuppressants [7, 14]. Niezgoda *et al.* reported a case where ulceration could not be stopped by corticosteroids alone, but after application of hyperbaric oxygen therapy, the disease progression was halted, and debridement with consecutive split skin grafting could be performed successfully. The authors were the first to describe postoperative use of vacuum-assisted closure that supported the complete healing of the lesion [15].

It must be considered that long-term exposure to corticosteroids or other immunosuppressive drugs puts patients at risk of developing life-threatening infections and other complications. Case reports have shown that the average treatment time can be reduced by surgical management of PG ulcers [9]. Summarizing other authors’ experiences, it can be inferred that surgery for PG lesions can reduce the duration of the disease without high risk for complications as long as the following principles are applied: ongoing immunosuppression, reduced disease activity, and soft tissue protective surgery.

The current concept for development of PG is of autoimmune etiology. Several cases in the literature did show bacterial growth on ulcers, but overall PG lesions are usually aseptic. This and the responsiveness to immune-suppressive therapy suggest that the theory of PG being caused by bacteria is obsolete [16, 17].

The role of antibiotics in PG treatment seems to be the prevention of complicating superinfections, particularly during systemic immunosuppression. Thus, most authors of PG case series use antibiotics in addition to anti-inflammatory drugs.

Adhering to the current recommendations in the literature regarding surgery for PG lesions and the case report of Long *et al.* [11], we were able to perform disease-unrelated surgery free of complications in our described patient. The retrospective study of Xia *et al*. [18] reported only a 15.1% occurrence of PG lesions after surgical procedures; thus, the PG-free course of our arthroplasty surgery cannot be attributed solely to our treatment regimen.

## Conclusions

Due to the rarity of this constellation, recommendations based on high-level evidence cannot be made for orthopedic trauma treatment during active PG. In our patient’s case, the use of pre- and perioperative steroids, combined with perioperative antibiotics and a soft tissue protective surgical technique, provided a successful strategy for the treatment of this rare challenge in orthopedic trauma surgery. This case report should encourage other surgeons facing this challenge not to avoid necessary operative treatment and improve patients’ quality of life by adhering to the described principles.

## Data Availability

Data sharing is not applicable to this article, because no datasets were generated or analyzed during the current study.

## Abbreviation

PG: Pyoderma gangrenosum
